# First person – Asli Adak and Romario Strasser

**DOI:** 10.1242/dmm.053235

**Published:** 2026-10-28

## Abstract

First Person is a series of interviews with the first authors of a selection of papers published in Disease Models & Mechanisms, helping researchers promote themselves alongside their papers. Asli Adak and Romario Strasser are co-first authors on ‘ Development of a laser injury model using human cardiac spheroids’, published in DMM. Asli conducted the research described in this article while a PhD student in Professor Nadia Mercader's lab at University of Bern, Bern, Switzerland. She is now a postdoctoral researcher in the lab of Professor Dr Camilla Schinner at University of Bern, investigating heart regeneration, development and diseases. Romario is a PhD student in the lab of Professor Nadia Mercader at University of Bern, investigating cardiac regeneration and development.

**Figure:**
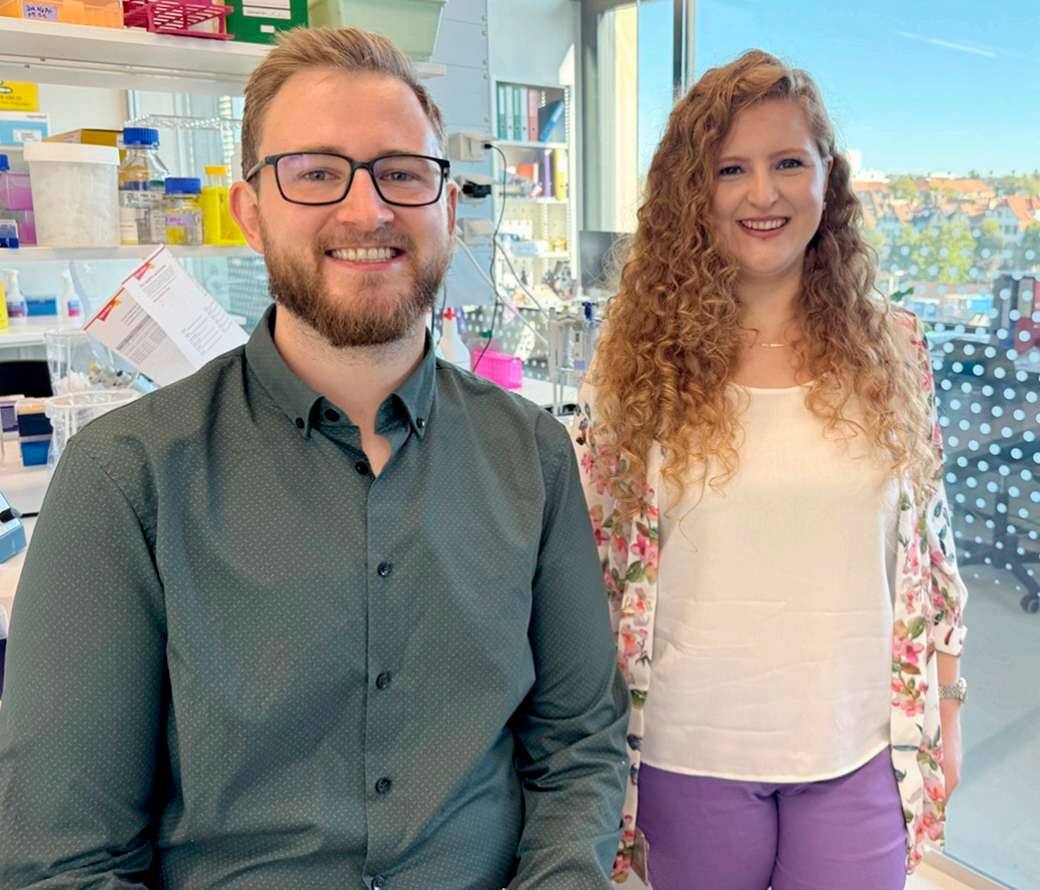
Asli Adak (right) and Romario Strasser (left)

**Who or what inspired you to become a scientist?**

**A.A.:** I have always been drawn to solving problems and discovering new things. This curiosity naturally led me to science, where I can explore and better understand the mechanisms underlying nature. Among the different fields of science, biology has always fascinated me because it offers the opportunity to understand how living systems function, adapt and interact. My desire to ask questions, explore the unknown and uncover new insights has continued to inspire me to pursue a career in science.

**R.S.:** I have always been fascinated by the natural sciences and the many mysteries of life and the universe. Discovering the ‘how’ and ‘why’ behind the processes we rely on every day brings me immense joy. To pursue this as a career and contribute – even in a small way – to uncovering these secrets is a dream come true.

We developed a new method to simulate heart damage in tiny, laboratory-grown human heart tissues by using a precise laser

**What is the main question or challenge in disease biology you are addressing in this paper? How did you go about investigating your question or challenge?**

**A.A./R.S.:** Acute myocardial infarction is a leading cause of death. Representative *in vitro* cardiac injury models based on human induced pluripotent stem cells (iPSCs) remain limited. Traditional 2D monocultures lack cellular diversity, microenvironmental mechanics and relevant injury physiology. Furthermore, physical injury methods like cryoinjury cannot be scaled down to microtissues. Focal laser ablation of 3D cardiac spheroids solves this, enabling precise, localized injury in a scalable, multicellular tissue model.

**How would you explain the main findings of your paper to non-scientific family and friends?**

**A.A./R.S.:** We developed a new method to simulate heart damage in tiny, laboratory-grown human heart tissues by using a precise laser. This helps us mimic what happens after a heart attack, so we can better understand how the heart heals itself and how we can help it recover.

**What are the potential implications of these results for disease biology and the possible impact on patients?**

**A.A./R.S.:** This method enables the scalable study of cardiac injury response and regeneration in human microtissues. Our model can be adapted for automated screening using reporter lines that provide rapid, quantifiable readouts. Additionally, the platform supports personalized medicine: iPSC-derived tissues can be generated from patient-specific cells or genetically edited cells via CRISPR-Cas9 to model distinct mutations. Unlike larger tissue models, this approach requires significantly fewer cells while maintaining a complex, multicellular architecture that remains expandable.

**Figure:**
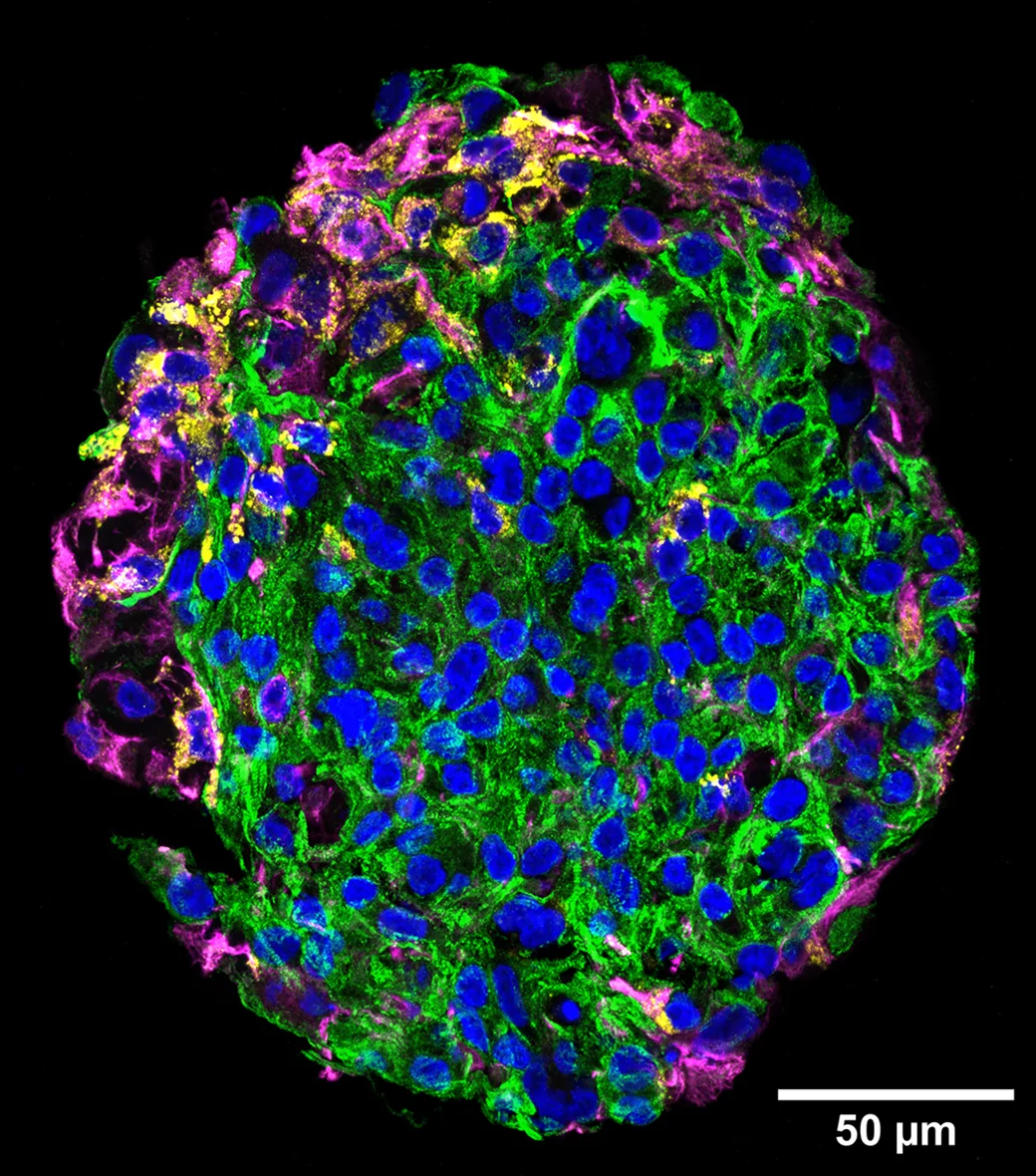
**Immunocytochemistry and confocal microscopy merged image of a cryosection of a laser-irradiated spheroid stained for vimentin (magenta), GFP (green), collagen-1α-1 (yellow) and DAPI (blue)**. Scale bar: 50 μm.

**Why did you choose DMM for your paper?**

**A.A./R.S.:** Given DMM's focus on insights into human disease mechanisms, we believe our project is a strong fit for the journal. We intend to use this injury model to investigate cardiac regeneration and fibrosis, with the ultimate goal of deepening our understanding of human cardiac repair and identifying potential new therapeutic approaches.

**Given your current role, what challenges do you face and what changes could improve the professional lives of other scientists in this role?**

**A.A./R.S.:** The biggest challenge is the pressure of the ‘publish or perish’ mindset, which values flashy results over the slow, careful work needed for true reproducibility. To improve life for early-career researchers, the academic system might benefit from focusing less on journal name dropping and instead reward thorough documentation, open methods and honest negative results.

**What's next for you?**

**A.A.:** I will pursue my research career in my current postdoctoral position, where I aim to investigate novel regulators of cardiac morphogenesis and cellular function in the heart using a stem cell-based, 3D human heart model combined with CRISPR screening.

**R.S.:** Continuing my PhD studies. I will now investigate cellular communication during heart regeneration by analysing generated knockout lines and how the knockout affects cardiomyocyte and cardiac fibroblast function and maturation.

The biggest challenge is the pressure of the ‘publish or perish’ mindset, which values flashy results over the slow, careful work needed for true reproducibility

**Tell us something interesting about yourself that wouldn't be on your CV**

**A.A.:** In my free time, I enjoy various forms of handcrafting, particularly embroidery, and I am currently learning how to sew. I also enjoy crocheting, as these activities allow me to be creative and express myself through different colours, patterns and designs. In addition to crafting, I have a strong interest in cooking and enjoy exploring new recipes from different cultures. Travelling is another passion of mine, particularly because it gives me the opportunity to experience different cultures and discover their local cuisines.

**R.S.:** I spend my free time either running Dungeons & Dragons campaigns as a Game Master or treating my kitchen like an experimental workshop. Whether I'm designing homebrew game mechanics or adjusting a baking recipe using reactor upscale factors and keeping experimental notes to refine a final product, I love taking complex systems apart, troubleshooting the variables, and building something creative, fun and tasty from scratch.
